# Editorial: Advances and perspectives in neuroplacentology

**DOI:** 10.3389/fendo.2023.1206072

**Published:** 2023-05-19

**Authors:** Claire-Marie Vacher, Alexandre Bonnin, Imran N. Mir, Anna A. Penn

**Affiliations:** ^1^ Department of Pediatrics, NewYork Presbyterian Hospital, New York, NY, United States; ^2^ Columbia University Irving Medical Center, Columbia University, New York, NY, United States; ^3^ Keck School of Medicine, University of Southern California, Los Angeles, CA, United States; ^4^ Department of Pediatrics, University of Texas Southwestern Medical Center, Dallas, TX, United States

**Keywords:** neuroplacentology, placenta, brain, preterm birth, neurodevelopmental disorders, preeclampsia, chorioamnionitis

The placenta is essential for pregnancy maintenance and fetal development. It regulates fetal growth by controlling the transport of oxygen, nutrients and waste products between the maternal and fetal circulation. It also serves as a protective and selective barrier filtering the passage of hormones, toxic agents and pathogens that could be harmful for the fetus. Recent studies have revealed a more specific role of the placenta in protecting and shaping the developing brain. The placenta produces a plethora of neuroactive hormones, growth factors, and immune molecules that can influence brain developmental trajectory, in region- and sex-dependent ways. Consequently, placental dysfunction or abruption may program the developing brain for long-term neurological and psychiatric morbidities. The growing body of evidence linking placental physiology and brain development has led to the emergence of a new field coined “neuroplacentology”. The current Research Topic explores different aspects of the latest research in neuroplacentology and offers new insights into the array of associations linking placental physiology and brain development.

Preterm birth refers to any live birth before 37 weeks of gestation. Premature delivery, which is marked by the premature loss of the placenta, exposes the immature cerebral tissue to adverse conditions that may compromise its development, lead to injury and potentially predispose the newborn to neurodevelopmental disorders, including a wide range of motor, cognitive, behavioral and emotional disorders (1). Preterm birth is a major public health issue that affects approximately 10% of the surviving newborns worldwide (2). The prematurity rate in the United States has not decreased substantially in recent decades, but the survival rate for preterm infants has improved. Eighty percent of infants weighing 500-1000 g will now survive, but with a significant risk of lifelong disabilities. Despite considerable improvements in neonatal care and therapies in recent years (3), preterm labor is still responsible for 70% of perinatal mortality and accounts for 50% of long-term neurobehavioral morbidities. The relative risks of these adverse outcomes are even higher for those born extremely prematurely (<28 weeks of gestation) (4). Placental conditions preceding preterm birth, such as intrauterine growth restriction, infection (chorioamnionitis) or preeclampsia, have also been shown to exacerbate the risk and severity of poor neurological outcomes caused by prematurity (5–8). Gardella et al. offer a comprehensive overview of this topic. The authors review and discuss different potential mechanisms linking compromised placental support and postnatal neurological outcomes in the context of fetal growth restriction and prematurity. White matter injuries are common complications of preterm birth, particularly in extremely preterm infants, due to the exposure to factors such as hypoxia, inflammation, oxidative stress and withdrawal of placental support (9–16). Marable et al. present an analysis of placental transcriptomes from a cohort of extremely low gestational age newborns (ELGANs) diagnosed with white matter injury. This study reveals that white matter damages among extremely preterm infants are associated with placental transcriptional signatures linked to endocrine disorders, metabolism, inflammation, immune response, and autism spectrum disorders.

Preeclampsia, a gestational hypertensive disorder occurring in 3 to 8% of pregnancies worldwide (17), is a leading cause of maternal and fetal morbidity and mortality. Preeclampsia is linked to clinical neurodevelopmental outcomes in children, including cognitive and psychiatric vulnerabilities, motor impairments, and stroke risk (18). It also increases the risk of fetal growth restriction and placental abruption, ~15% of preterm deliveries being due to preeclampsia (19). There is a pressing need to understand the mechanisms leading to the neonatal outcomes of preeclampsia because there are no well-established measures for primary prevention, delivery remaining the ultimate treatment. The etiology of preeclampsia is not fully understood, but recent research suggests a contributing role of placentally derived angiogenic factor release into the maternal circulation. In particular, an imbalance between PlGF (Placental Growth Factor) and sFlt-1 (soluble Fms-like tyrosine kinase-1) has been associated with the onset of the disorder (20, 21). Interestingly, higher sFlt-1/PlGF ratio in the maternal serum has also been linked to higher risk of fetal growth restriction, preterm labor, and reduced APGAR (Appearance, Pulse, Grimace, Activity and Respiration) score (which assesses for signs of hemodynamic compromise at birth) (22). However, the predictive value of sFlt-1/PlGF ratio for neurological vulnerabilities of prematurity has never been evaluated. In this Research Topic, Middendorf et al. show, in a cohort of 88 preterm infants, that low birth weight was associated with increased maternal sFlt-1/PlGF ratio and worse motor score (as measured by Motor Optimality Score-Revised, MOS-R). However, no direct correlation between sFlt-1/PlGF ratio and motor impairments was found. Evaluating the developmental progress of the cohort through follow-up assessments may provide additional insights into the long-term outcomes of the preterm infants beyond their initial assessment.

The placenta plays a critical role in regulating the *in-utero* immunological state during pregnancy. It helps maintaining the balance between an active immune response against potential intrauterine infections and an immunosuppression that preserves semi-allogeneic fetal development. This optimal balance between pro- and anti-inflammatory signals in the intrauterine milieu is temporally regulated by the placental secretion of cytokines (23, 24), which can influence fetal brain development (25). Infection of the placenta and its membranes, known as chorioamnionitis, is a major cause of preterm delivery (26–29), and may place the fetus at risk for long-term neurological outcomes (30–40). In the current Research Topic, Leon et al. report elevated rates of histological signs of placental inflammation in term infants with perinatal stroke. This finding represents a substantial advancement in our knowledge of the perinatal stroke etiology. The authors stress the importance of continued research in prenatal assessment of placental health, using biomarker signatures or magnetic resonance imaging, to identify perinatal stroke risk early and initiate interventions when possible. Chorioamnionitis is characterized by elevated pro-inflammatory cytokines such as interleukin-1β (IL-1β) (41), leading to excessive inflammatory processes and subsequent fetal brain injury (42). In an experimental model of chorioamnionitis induced by Group B Streptococcus (GBS), Ayash et al. demonstrate that the targeted blockade of IL-1β activity using IL-1a receptor antagonist (IL-1Ra) alleviates placental inflammation and the resulting Fetal Inflammatory Response Syndrome (FIRS). Furthermore, prenatal IL-1 blockade reduces or prevents some of the neurobehavioral impairments resulting from FIRS in the female offspring, but not in males. These findings open new avenues on the therapeutic potential of IL-1Ra to prevent some of the neurobehavioral alterations resulting from placental inflammatory diseases. This study also highlights the importance of considering biological sex when studying the mechanisms that contribute to the etiology, manifestation and treatment of neurodevelopmental disorders.

The increasing prevalence of obesity among women of child-bearing age has been an issue of growing concern in recent years, with studies showing that it may contribute to an elevated risk of pregnancy complications (43). This is particularly true for women of lower socioeconomic status and certain racial groups, with socio-demographic features and stress being identified as potential factors (44–46). Among Black women, higher rates of premature births and small-for-gestational age infants have been reported (2), which may in part be linked to placental conditions. In this context, the study by Williams et al. sheds new light on the complex interplay between maternal health markers (i.e., body mass index and inflammation), race, placental function, and infant outcomes. These findings have important implications for the development of targeted interventions aimed at reducing modifiable pre-pregnancy factors, such as body mass index (BMI) or stress, to reduce pregnancy complications that can ultimately improve maternal and infant health outcomes.

The placenta not only exposes the fetus to negative signals in pathological conditions, but also produces protective factors. A growing body of evidence indicates that the placenta supports fetal brain development through the release of neuroactive and neuroprotective signaling molecules (47–51). Allopregnanolone (ALLO) is a neuroactive metabolite of progesterone (52) that acts as a potent positive allosteric modulator of the GABA_A_ receptors (GABA_A_-Rs) (53). In the fetal brain, ALLO levels peak in mid-to-late gestation and are greater than at any other period in life due to a high placental production (54). ALLO administration has been shown to exert neurotrophic, neuroprotective and anti-inflammatory actions in a number of experimental models of neurological conditions, including perinatal brain injury (55). Placental ALLO insufficiency has been associated with altered brain myelination and male-specific autistic-like behavior in a conditional knockout mouse model (51). Using the same model, Bakalar et al. further show that the lack of placental ALLO disrupts corticogenesis and female somatosensory function. These findings suggest that placental neurosteroids regulate fetal brain development and long-term behavior differently in males and females. Placental hormones may thus target specific structures, circuits and cells, and deviation from physiological conditions might have sex-biased, enduring neurobehavioral consequences.

In conclusion, this Research Topic provides new insights into the mechanisms underlying the placental origin of short and long-term neurological disorders. This special issue shows that the risk and degree of placental and brain disorders are influenced by a complex interplay of factors, such as the timing of insult, biological sex and social determinants of maternal health. Identifying placental risk factors for perinatal brain injuries and neurodevelopmental impairments represents a central aspect of the research in neuroplacentology. The latest advances in this topic highlight the importance of evaluating circulating placental factors, placental histology and transcriptional signatures to identify the individuals at risk and mitigate the impact of injury on fetal brain, through the development of new interventions and preventive strategies. In this regard, the development of more advanced, non-invasive tools and sensors is needed to monitor placental development and detect prenatal anomalies, allowing for timely interventions.

## Author contributions

C-MV supervised and wrote the editorial article. AB, IM and AP reviewed and approved the editorial manuscript.

## References

[1] Volpe JJ. The encephalopathy of prematurity–brain injury and impaired brain development inextricably intertwined. Semin Pediatr Neurol (2009) 16:167–78. doi: 10.1016/j.spen.2009.09.005 PMC2799246 19945651

[2] Dimes M.o. Premature birth report card (2022). Available at: https://www.marchofdimes.org/peristats/reports/united-states/report-card.

[3] Wilson-Costello D Friedman H Minich N Siner B Taylor G Schluchter M. Improved neurodevelopmental outcomes for extremely low birth weight infants in 2000-2002. Pediatrics (2007) 119:37–45. doi: 10.1542/peds.2006-1416 17200269

[4] Hirschberger RG Kuban KCK O’Shea TM Joseph RM Heeren T Douglass LM. Co-Occurrence and severity of neurodevelopmental burden (Cognitive impairment, cerebral palsy, autism spectrum disorder, and epilepsy) at age ten years in children born extremely preterm. Pediatr Neurol (2018) 79:45–52. doi: 10.1016/j.pediatrneurol.2017.11.002 29310907 PMC5803305

[5] Bennet L Dhillon S Lear CA van den Heuij L King V Dean JM. Chronic inflammation and impaired development of the preterm brain. J Reprod Immunol (2018) 125:45–55. doi: 10.1016/j.jri.2017.11.003 29253793

[6] Chau V McFadden DE Poskitt KJ Miller SP. Chorioamnionitis in the pathogenesis of brain injury in preterm infants. Clin Perinatol (2014) 41:83–103. doi: 10.1016/j.clp.2013.10.009 24524448

[7] Garfinkle J Miller S. The placenta and neurodevelopment in preterm newborns. NeoReviews (2018) 19(8):e456–66. doi: 10.1542/neo.19-8-e456

[8] Warshafsky C Pudwell J Walker M Wen SW Smith GN Preeclampsia New Emerging T. Prospective assessment of neurodevelopment in children following a pregnancy complicated by severe pre-eclampsia. BMJ Open (2016) 6:e010884. doi: 10.1136/bmjopen-2015-010884 PMC4947739 27388354

[9] Burd I Chai J Gonzalez J Ofori E Monnerie H Le Roux PD. Beyond white matter damage: fetal neuronal injury in a mouse model of preterm birth. Am J Obstet Gynecol (2009) 201:279 e1–8. doi: 10.1016/j.ajog.2009.06.013 PMC2740757 19733279

[10] Gilles FH Leviton A. Neonatal white matter damage and the fetal inflammatory response. Semin Fetal Neonatal Med (2020) 25:101111. doi: 10.1016/j.siny.2020.101111 32299712

[11] Korzeniewski SJ Romero R Cortez J Pappas A Schwartz AG Kim CJ. A “multi-hit” model of neonatal white matter injury: cumulative contributions of chronic placental inflammation, acute fetal inflammation and postnatal inflammatory events. J Perinat Med (2014) 42:731–43. doi: 10.1515/jpm-2014-0250 PMC5987202 25205706

[12] Kuypers E Ophelders D Jellema RK Kunzmann S Gavilanes AW Kramer BW. White matter injury following fetal inflammatory response syndrome induced by chorioamnionitis and fetal sepsis: lessons from experimental ovine models. Early Hum Dev (2012) 88:931–6. doi: 10.1016/j.earlhumdev.2012.09.011 23078831

[13] Levesque ML Fahim C Ismaylova E Verner MP Casey KF Vitaro F. The impact of the *in utero* and early postnatal environments on grey and white matter volume: a study with adolescent monozygotic twins. Dev Neurosci (2015) 37:489–96. doi: 10.1159/000430982 26279175

[14] Massaro AN Evangelou I Fatemi A Vezina G McCarter R Glass P. White matter tract integrity and developmental outcome in newborn infants with hypoxic-ischemic encephalopathy treated with hypothermia. Dev Med Child Neurol (2015) 57:441–8. doi: 10.1111/dmcn.12646 PMC4543365 25492527

[15] Perlman JM. White matter injury in the preterm infant: an important determination of abnormal neurodevelopment outcome. Early Hum Dev (1998) 53:99–120. doi: 10.1016/S0378-3782(98)00037-1 10195704

[16] Young JM Vandewouw MM Morgan BR Smith ML Sled JG Taylor MJ. Altered white matter development in children born very preterm. Brain Struct Funct (2018) 223(5):2129-41. doi: 10.1007/s00429-018-1614-4 29380120

[17] Karrar SA Hong PL. Preeclampsia. In: StatPearls. Treasure Island (FL): StatPearls Publishing (2023).

[18] Gumusoglu SB Chilukuri ASS Santillan DA Santillan MK Stevens HE. Neurodevelopmental outcomes of prenatal preeclampsia exposure. Trends Neurosci (2020) 43:253–68. doi: 10.1016/j.tins.2020.02.003 PMC7170230 32209456

[19] Roberts JM Pearson GD Cutler JA Lindheimer MD National Heart L Blood I. Summary of the NHLBI working group on research on hypertension during pregnancy. Hypertens Preg (2003) 22:109–27. doi: 10.1081/PRG-120016792 12908996

[20] Allen RE Rogozinska E Cleverly K Aquilina J Thangaratinam S. Abnormal blood biomarkers in early pregnancy are associated with preeclampsia: a meta-analysis. Eur J Obstet Gynecol Reprod Biol (2014) 182:194–201. doi: 10.1016/j.ejogrb.2014.09.027 25305662

[21] Cerdeira AS Agrawal S Staff AC Redman CW Vatish M. Angiogenic factors: potential to change clinical practice in pre-eclampsia? BJOG (2018) 125:1389–95. doi: 10.1111/1471-0528.15042 PMC6175139 29193681

[22] Chang YS Chen CN Jeng SF Su YN Chen CY Chou HC. The sFlt-1/PlGF ratio as a predictor for poor pregnancy and neonatal outcomes. Pediatr Neonatol (2017) 58:529–33. doi: 10.1016/j.pedneo.2016.10.005 28571908

[23] Hussain T Murtaza G Kalhoro DH Kalhoro MS Yin Y Chughtai MI. Understanding the immune system in fetal protection and maternal infections during pregnancy. J Immunol Res (2022) 2022:7567708. doi: 10.1155/2022/7567708 35785037 PMC9249541

[24] Mor G Cardenas I Abrahams V Guller S. Inflammation and pregnancy: the role of the immune system at the implantation site. Ann N Y Acad Sci (2011) 1221:80–7. doi: 10.1111/j.1749-6632.2010.05938.x PMC3078586 21401634

[25] Yockey LJ Iwasaki A. Interferons and proinflammatory cytokines in pregnancy and fetal development. Immunity (2018) 49:397–412. doi: 10.1016/j.immuni.2018.07.017 30231982 PMC6152841

[26] Afkham A Eghbal-Fard S Heydarlou H Azizi R Aghebati-Maleki L Yousefi M. Toll-like receptors signaling network in pre-eclampsia: an updated review. J Cell Physiol (2019) 234:2229–40. doi: 10.1002/jcp.27189 30221394

[27] Aldo PB Krikun G Visintin I Lockwood C Romero R Mor G. A novel three-dimensional *in vitro* system to study trophoblast-endothelium cell interactions. Am J Reprod Immunol (2007) 58:98–110. doi: 10.1111/j.1600-0897.2007.00493.x 17631003 PMC7062291

[28] Goldenberg RL Hauth JC Andrews WW. Intrauterine infection and preterm delivery. N Engl J Med (2000) 342:1500–7. doi: 10.1056/NEJM200005183422007 10816189

[29] Rodrigues-Duarte L Pandya Y Neres R Penha-Goncalves C. Fetal and maternal innate immunity receptors have opposing effects on the severity of experimental malaria in pregnancy: beneficial roles for fetus-derived toll-like receptor 4 and type I interferon receptor 1. Infect Immun 86 (2018). doi: 10.1128/IAI.00708-17 PMC5913849 29440369

[30] Agrawal V Hirsch E. Intrauterine infection and preterm labor. Semin Fetal Neonatal Med (2012) 17:12–9. doi: 10.1016/j.siny.2011.09.001 PMC3242863 21944863

[31] Blencowe H Cousens S Chou D Oestergaard M Say L Moller AB. Born too soon: the global epidemiology of 15 million preterm births. Reprod Health (2013) 10 Suppl 1:S2. doi: 10.1186/1742-4755-10-S1-S2 24625129 PMC3828585

[32] Blencowe H Cousens S Oestergaard MZ Chou D Moller AB Narwal R. National, regional, and worldwide estimates of preterm birth rates in the year 2010 with time trends since 1990 for selected countries: a systematic analysis and implications. Lancet (2012) 379:2162–72. doi: 10.1016/S0140-6736(12)60820-4 22682464

[33] Deguchi K Mizuguchi M Takashima S. Immunohistochemical expression of tumor necrosis factor alpha in neonatal leukomalacia. Pediatr Neurol (1996) 14:13–6. doi: 10.1016/0887-8994(95)00223-5 8652010

[34] Kadhim H Tabarki B Verellen G De Prez C Rona AM Sebire G. Inflammatory cytokines in the pathogenesis of periventricular leukomalacia. Neurology (2001) 56:1278–84. doi: 10.1212/WNL.56.10.1278 11376173

[35] Kidokoro H Anderson PJ Doyle LW Woodward LJ Neil JJ Inder TE. Brain injury and altered brain growth in preterm infants: predictors and prognosis. Pediatrics (2014) 134:e444–53. doi: 10.1542/peds.2013-2336 25070300

[36] Lamont RF Sawant SR. Infection in the prediction and antibiotics in the prevention of spontaneous preterm labour and preterm birth. Minerva Ginecol (2005) 57:423–33. doi: 10.1046/j.1471-0528.2003.00034.x 16170287

[37] Oliver RS Lamont RF. Infection and antibiotics in the aetiology, prediction and prevention of preterm birth. J Obstet Gynaecol (2013) 33:768–75. doi: 10.3109/01443615.2013.842963 24219711

[38] Ortinau C Neil J. The neuroanatomy of prematurity: normal brain development and the impact of preterm birth. Clin Anat (2015) 28:168–83. doi: 10.1002/ca.22430 25043926

[39] Romero R Gomez R Chaiworapongsa T Conoscenti G Kim JC Kim YM. The role of infection in preterm labour and delivery. Paediatr Perinat Epidemiol (2001) 15 Suppl 2:41–56. doi: 10.1046/j.1365-3016.2001.00007.x 11520399

[40] Yoon BH Romero R Park JS Kim CJ Kim SH Choi JH. Fetal exposure to an intra-amniotic inflammation and the development of cerebral palsy at the age of three years. Am J Obstet Gynecol (2000) 182:675–81. doi: 10.1067/mob.2000.104207 10739529

[41] Owen JC Garrick SP Peterson BM Berger PJ Nold MF Sehgal A. The role of interleukin-1 in perinatal inflammation and its impact on transitional circulation. Front Pediatr (2023) 11:1130013. doi: 10.3389/fped.2023.1130013 36994431 PMC10040554

[42] Liu D Gao Q Wang Y Xiong T. Placental dysfunction: the core mechanism for poor neurodevelopmental outcomes in the offspring of preeclampsia pregnancies. Placenta (2022) 126:224–32. doi: 10.1016/j.placenta.2022.07.014 35872512

[43] Giouleka S Tsakiridis I Koutsouki G Kostakis N Mamopoulos A Kalogiannidis I. Obesity in pregnancy: a comprehensive review of influential guidelines. Obstet Gynecol Surv (2023) 78:50–68. doi: 10.1097/OGX.0000000000001091 36607201

[44] Braveman P Dominguez TP Burke W Dolan SM Stevenson DK Jackson FM. Explaining the black-white disparity in preterm birth: a consensus statement from a multi-disciplinary scientific work group convened by the march of dimes. Front Reprod Health (2021) 3:684207. doi: 10.3389/frph.2021.684207 36303973 PMC9580804

[45] Heslehurst N. Identifying a’t risk’ women and the impact of maternal obesity on national health service maternity services. Proc Nutr Soc (2011) 70:439–49. doi: 10.1017/S0029665111001625 21854697

[46] Luke B. Adverse effects of female obesity and interaction with race on reproductive potential. Fertil Steril (2017) 107:868–77. doi: 10.1016/j.fertnstert.2017.02.114 PMC9761479 28366413

[47] Bonnin A Goeden N Chen K Wilson ML King J Shih JC. A transient placental source of serotonin for the fetal forebrain. Nature (2011) 472:347–50. doi: 10.1038/nature09972 PMC3084180 21512572

[48] Bonnin A Levitt P. Placental source for 5-HT that tunes fetal brain development. Neuropsychopharmacology (2012) 37:299–300. doi: 10.1038/npp.2011.194 22157865 PMC3238076

[49] Bonnin A Torii M Wang L Rakic P Levitt P. Serotonin modulates the response of embryonic thalamocortical axons to netrin-1. Nat Neurosci (2007) 10:588–97. doi: 10.1038/nn1896 17450135

[50] Fitzgerald E Hor K Drake AJ. Maternal influences on fetal brain development: the role of nutrition, infection and stress, and the potential for intergenerational consequences. Early Hum Dev (2020) 150:105190. doi: 10.1016/j.earlhumdev.2020.105190 32948364 PMC7481314

[51] Vacher CM Lacaille H O’Reilly JJ Salzbank J Bakalar D Sebaoui S. Placental endocrine function shapes cerebellar development and social behavior. Nat Neurosci (2021). doi: 10.1038/s41593-021-00896-4 PMC8481124 34400844

[52] Paul SM Purdy RH. Neuroactive steroids. FASEB J (1992) 6:2311–22. doi: 10.1096/fasebj.6.6.1347506 1347506

[53] Paul SM Pinna G Guidotti A. Allopregnanolone: from molecular pathophysiology to therapeutics. A historical perspect Neurobiol Stress (2020) 12:100215. doi: 10.1016/j.ynstr.2020.100215 PMC7231972 32435665

[54] Nguyen PN Yan EB Castillo-Melendez M Walker DW Hirst JJ. Increased allopregnanolone levels in the fetal sheep brain following umbilical cord occlusion. J Physiol (2004) 560:593–602. doi: 10.1113/jphysiol.2004.069336 15331682 PMC1665267

[55] Guennoun R Labombarda F Gonzalez Deniselle MC Liere P De Nicola AF Schumacher M. Progesterone and allopregnanolone in the central nervous system: response to injury and implication for neuroprotection. J Steroid Biochem Mol Biol (2015) 146:48–61. doi: 10.1016/j.jsbmb.2014.09.001 25196185

